# Supplementary material for: Phytoestrogen Coumestrol Selectively Inhibits Monoamine Oxidase-A and Amyloid β Self-Aggregation
Source: Nutrients. 2022 Sep 16;14(18):3822. doi: 10.3390/nu14183822 (PMC9502896; doi:10.3390/nu14183822)
Supplement: Supplementary file 1 [file nutrients-14-03822-s001.zip › nutrients-1924691-supplementary.pdf]

Table S1. Kinetic parameters for Lineweaver-Burk plot and its secondary plots.

[Substrate, μM]	[Coumestrol, μM]	RLU					1/V [= 1/(RLU/60min)]	
		1	2	3	Mean	SD	Mean	SD
160	0	465715	468709	486780	473734.7	11396.339	0.000127	0.000003
	2.5	219596	233409	236873	229959.3	9140.505	0.000261	0.000011
	10	118306	128367	117253	121308.7	6135.3284	0.000495	0.000024
	20	69778	76748	80190	75572	5304.6836	0.000797	0.000057
80	0	404077	393284	404201	400520.7	6267.4438	0.000150	0.000002
	2.5	126502	126966	125654	126374	665.29993	0.000475	0.000003
	10	66093	64057	61882	64010.67	2105.8823	0.000938	0.000031
	20	36461	36478	40008	37649	2042.9716	0.001597	0.000084
40	0	332626	318019	317481	322708.7	8592.8742	0.000186	0.000005
	2.5	83446	79324	82581	81783.67	2173.5976	0.000734	0.000020
	10	33297	39254	37597	36716	3074.6679	0.001642	0.000142
	20	20692	20299	20131	20374	287.92186	0.002945	0.000041

Table S2. Fluorescent signals obtained from self Aβ_{25-35} aggregation assay.

Purpose	Compounds	Concentrations (μM)	Fluorescence (Emission = 490 nm / Excitation = 446 nm, *n* = 3)			
			1	2	3	Control for sample
Screening active compound	Blank	-	274.456	273.691	267.102	269.222
	Control	-	826.227	807.578	779.828	325.1847
	Rutin	100	556.703	574.097	523.388	308.423
	Daidzein	100	752.714	744.841	739.542	436.703
	Genistein	100	656.961	678.75	636.556	345.35
	Coumestrol	100	420.995	408.908	418.89	323.923
	Curcumin	20	446.557	456.907	436.435	319.674
Dose-dependent curves	Coumestrol	5	874.9012	872.4322	899.9266	307.558
		10	820.955	835.446	846.767	321.491
		20	725.8814	709.3678	704.8239	320.227
		50	578.864	549.001	576.214	310.912

		100	484.285	460.697	486.519	315.877
	Curcumin	5	714.733	699.957	732.987	309.277
		10	659.3354	647.8281	645.7345	305.887
		20	508.323	494.704	529.05	287.149
	Control	-	980.052	989.522	1015.874	319.08
